# Vincristine liposomes with smaller particle size have stronger diffusion ability in tumor and improve tumor accumulation of vincristine significantly

**DOI:** 10.18632/oncotarget.20162

**Published:** 2017-08-10

**Authors:** Siyu Ma, Mingyuan Li, Nan Liu, Ying Li, Zhiping Li, Yang Yang, Fanglin Yu, Xiaoqin Hu, Cheng Liu, Xingguo Mei

**Affiliations:** ^1^ Institute of Pharmacology and Toxicology, Academy of Military Medical Sciences, Beijing, PR China; ^2^ Wuhan Institute of Technology, Wuhan, PR China; ^3^ China International Science and Technology Cooperation Base of Food Nutrition/Safety and Medicinal Chemistry, Sino-French Joint Lab of Food Nutrition/Safety and Medicinal Chemistry, Key Laboratory of Industrial Fermentation Microbiology of Ministry of Education, Tianjin Key Laboratory of Industry Microbiology, College of Biotechnology, Tianjin University of Science and Technology, Tianjin, PR China

**Keywords:** particle size, diffusion ability, targeting effect, liposome, tumor targeting

## Abstract

The passive targeting is the premise of active targeting that could make nanocarrier detained in tumor tissue. The particle size is the most important factor that influences the diffusion and distribution of nanoparticle both *in vivo* and *in vitro*. In order to investigate the relationship between particle size and diffusion ability, two kinds of liposome loaded with Vincristine (VCR-Lip) were prepared. The diffusion behavior of VCR-Lip with different particle size and free VCR was compared through diffusion stability study. The diffusion ability from 12-well culture plate to Millipore transwell of each formulation reflected on HepG-2 cytotoxicity results. Different cell placement methods and drug adding positions were used to study the VCR-Lip diffusion behaviors, which influenced the apoptosis of HepG-2 cell. The different cell uptake of Nile red–Lip and free Nile red was compared when changed the adding way of fluorescent fluorescein. To study the penetration ability in HepG-2 tumor spheroids, we constructed 30 nm and 100 nm Cy5.5-Lip to compare with free Cy5.5. Then the anti-tumor effect, tissue distribution of free VCR injection, 30 nm and 100 nm VCR-Lip were further investigated on the HepG-2 tumor bearing nude mice. The results of these study showed that the diffusion ability of free drug and fluorescent fluorescein was remarkable stronger than which encapsulated in liposomes. Moreover, diffusion ability of smaller liposome was stronger than larger one. In this way, 30 nm liposome had not only faster and stronger tumor distribution than 100 nm liposome, but also higher tumor drug accumulation than free drug as well. Our study provided a new thinking to improve the targeting efficiency of nano drug delivery system, no matter passive or active targeting.

## INTRODUCTION

In recent years, overwhelming majority of the studies on targeting drug delivery system [1] are focus on seeking new target [2,3], new formulation [4], new material [5,6] or more special modification [7,8] to improve the targeting effect. Especially for the active targeting agent [9], this aims to enhance cellular uptake by ligands modification on the surface of nanocarrier [10]. However, the actual effects of numerous studies do not significantly improve the targeting efficiency [11]. In spite of decades of investigation, there is no active targeting nanocarrier has come into the market yet, while molecular targeting has achieved great success. One of the reasons is that they ignored the passive targeting is the basic premise of the nanocarrier taking drugs into the tumor tissue [12]. And the particle size [13,14] of nanocarrier is one of the most important factors influencing the passive targeting [15], because that particle sizes could influence the *in vivo* behavior of nanocarrier. Generally, the anti-tumor drugs or corresponding nanocarrier were administrated by intravenous injection or intravenous infusion [16]. After getting into circulation, some drugs will bind with the plasma protein (5nm) [17] and the other free drugs will quickly diffuse into tissue, and then diffuse into the space surrounding target cells. The drugs could take the therapeutic effect only when having the opportunity to contact with the cells [18,19]. According to the diffusion flux formula (1) [20] and the Stokes-Einstein Equation (2) [21,22], we can see that the diffusion ability of nanocarrier is the most important factor to improve their antitumor effect.

$$J=-D\frac{\text{dc}}{\text{dx}}$$ (1)

Where J is diffusion flux which mean the quantity of material diffusing through the unit area per unit time, D is the diffusion coefficient of particles, $\frac{d_{c}}{d_{x}}$ is the concentration gradient, the minus sign representative the diffusion direction is inverse concentration gradient.

$$D=\frac{\text{RT}}{L}\times \frac{1}{6\pi \eta r}$$ (2)

Where R is the Boltzmann's constant, T is the absolute temperature, L is the Avogadro's number; η is the dynamic viscosity of solution, r is the radius of the spherical particle.

From these formulas above, the particle size increase will reduce the diffusion coefficient and concentration gradient. They are proportional to the diffusion flux. The nanocarrier must be firstly taken into tumor vessels by blood circulation before they enter into the tumor tissue [23]. Although there are large numbers of wide fenestration on the tumor angiogenesis [24], the nanocarriers have to overcome the elevated interstitial fluid pressure [25] and the sticky extracellular matrix [26]. All of these will weaken the diffusion effect of nanoparticles. On the other hand, the high drug loading of nanocarrier means the total number of carriers (concentration) will be reduced compare to free drug. Moreover, the diffusion coefficient of nanocarrier is much less than the drug molecular. All of these leads to less number of nanocarrier could diffuse into the tumor tissue through tumor vessels. In other words, even for the modified nanocarriers, they also depend on chance to interact with the complementary recipient and to play the active targeting role. We performed a series of related studies *in vitro* and *in vivo* to prove our hypothesis that reducing the particle size could improve tumor targeting efficient of nanocarrier.

## RESULTS AND DISCUSSION

### Characteristics of liposome

**Figure 1:**
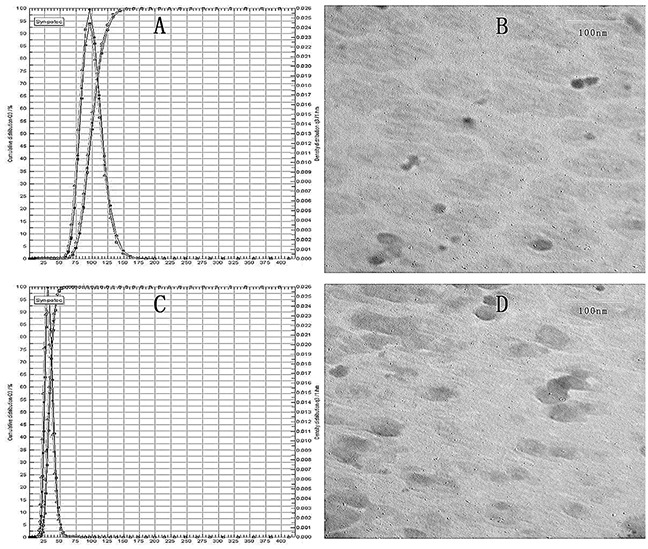
The Particle distribution and morphology of VCR-Lip **(A)**&**(C)** Particle size distribution. **(B)**&**(D)** TEM images of two particle size of VCR-Lip.

100 nm and 30 nm liposomal formulations containing VCR were prepared by the film dispersion method [27,28] and reverse evaporation method [29], respectively. The particle size was determined by the laser particle analyzer and morphologically characterized by TEM. As Figure 1A and 1C showed, the large particle VCR-Lips was 100.0±2.5 nm and the small was 30.0±1.6 nm. The result also showed that the particle size measured by two different methods were consistent. The drug loading and encapsulation efficiency is summarized in Table 1. The VCR encapsulation efficiency of 100 nm VCR-Lips was 96.28±2.38% and the 30 nm ones was 94.73±5.26%. And the drug loading of two kinds of VCR-Lips was 1.70±0.12 mg/mL and 1.59±0.46 mg/mL, respectively.

**Table 1:** The entrapment efficiency and drug loading of two particle size VCR-Lip (n=3)

Particle size (nm)	Entrapment efficiency (%)	Drug loading(mg/mL)
30.0±1.6	94.73±5.26	1.59±0.46
100.0±2.5	96.28±2.38	1.70±0.12

### The turbiscan stability index of free VCR, 100 nm and 30 nm VCR-Lip

**Figure 2:**
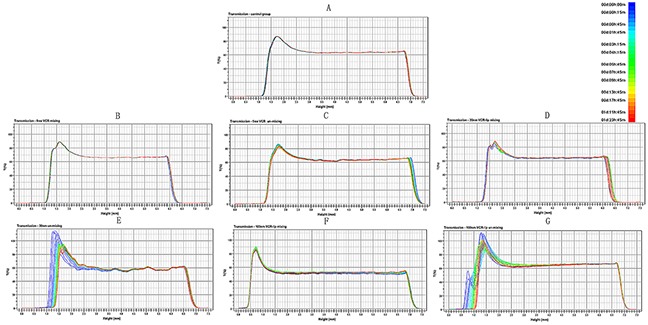
The process of preparation diffusion when dilution with PBS **(A)**. The PBS blank control. **(B)**&**(C)** The mixing and unmixed of free-VCR. **(D)**&**(E)** The mixing and unmixed of 30nm VCR-Lip. **(F)**&**(G)** The mixing and unmixed of 100nm VCR-Lip.

**Figure 3:**
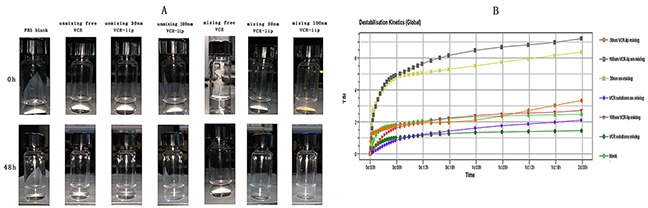
The turbiscan stability of free VCR, 100 nm and 30 nm VCR-Lip **(A)** The diffusion state of different VCR-Lip and free VCR in turbiscan sample bottles. **(B)** The destabilization kinetics of each mixing and unmixed group.

We used the turbiscan Lab^R^ Expert to evaluate the diffusion ability of different formulations *in vitro*. Figure 2B-2G was the transmission profiles when diluted the free VCR and 30 nm VCR-Lip, 100 nm VCR-Lip formulation with mixed and unmixed method. The PBS (Figure 2A) was the blank control for other groups. The results showed that each formulation of mixing group (Figure 2B, 2D, and 2F) was no apparent aggregation or sedimentation during the scan. However, the un-mixing group showed that 100 nm VCR-Lip (Figure 2G) had the floating phenomenon in the early stage and sedimentation in the later period during the scanning, which mean that the larger particle size liposome could not spontaneously diffusion uniformity in 48 h. The 30 nm VCR-Lip (Figure 2E) could diffuse uniformity when the scan was completed. For the free VCR solution (Figure 2C), it could rapidly reach the diffusion equilibrium. Figure 3B was the destabilization kinetics of each group. The value of destabilization kinetics could give the quantification of the diffusion equilibrium state during the scanning, the smaller value of destabilization kinetics meant that the time dilution process reach the diffusion equilibrium was shorter. The 100 nm and 30 nm VCR-Lip un-mixing group have larger destabilization kinetics compared to the free VCR un-mixing group and blank control group, moreover this value increased when the particle size was larger. This prompted that the larger particle size of liposome, more difficult to diffusion equilibrium *in vitro*. For the destabilization kinetics of mixing groups was close to the blank control group and the free VCR solution group which had the minor value. Destabilization kinetics of mixing and un-mixing free VCR solution groups was consistent to the blank control group of PBS. This result showed that the VCR solution could get diffusion equilibrium faster than the nano formulation. For smaller size nano formulation, the time to achieve balance was shorter than the larger one. Figure 3A showed the turbiscan sample bottles containing the diluted sample of each groups at the 0 h and 48 h. For the mixing groups of free VCR solution, 30 nm VCR-lip, 100 nm VCR-lip and the un-mixing groups of VCR solution are uniform during scanning process in the turbiscan Lab^R^ Expert for 48 h. The 30 nm VCR-Lip of un-mixing group could reach the diffusion equilibrium after place for 48 h. However, for the 100 nm un-mixing VCR-Lip group, it still did not reach diffusion equilibrium which had obvious stratification at 48 h. This result meant that the larger liposome could not spontaneously diffuse uniform without external force. This could preliminary inference that the larger liposome maybe difficult to diffuse into the internal tumor tissue from the tumor vessels *in vivo*, because of the slow liquid flow formed the blood capillary in the tissues.

### The effect of diffusion ability on cytotoxicity

The membrane pore at bottom of Millipore transwell is about 400 nm. For 30 nm and 100 nm VCR-Lips, they were easy to pass through it.

**Figure 4:**
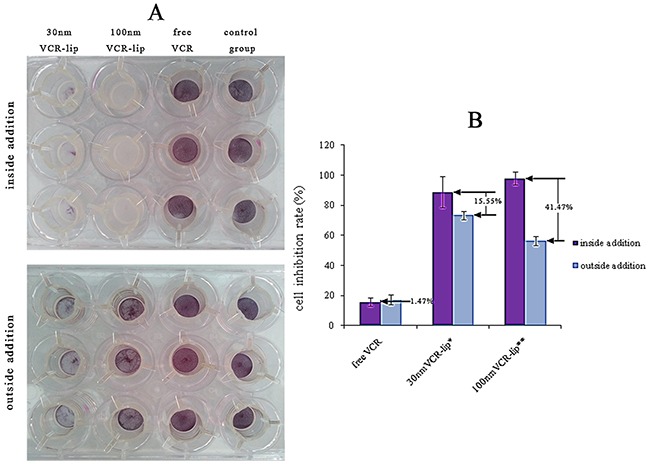
The different of preparation and drug added methods influence the cytotoxicity **(A)** The cell survival rate in Millipore transwell after added drug for 24 h. **(B)** The cell inhibition rate of each group.

To study the diffusion ability of free VCR solution, 30 nm and 100 nm VCR-Lips pass through the membrane [30], these different formulations were respectively added to the inside of Millipore transwell and the 12 well-plate which was at the outside of Millipore transwell. The result was showed in the Figure 4A and 4B, which was MTT staining of the HepG-2 cells in the Millipore transwell after the cells were adopted by different VCR formulations and different VCR formulations added methods. The cell inhibition rate of the inside and outside addition of free VCR was respective 15.86±2.67% and 17.33±3.32% (P>0.05). For 30 nm VCR-Lip, the cell inhibition rate of inside addition was 88.94±10.50% and outside addition was 73.38 ±2.80% (P<0.05). The inside addition and outside addition of 100 nm VCR-Lip was 97.96±4.35% and 56.49±3.14% (p<0.01), respectively. The result showed that no matter which addition method, free VCR solution had similar cytotoxicity, for because of the VCR molecular could diffuse pass membrane fenestrate and become uniform dispersed very quickly. For the VCR-Lip, the different cytotoxicity between the drug added way was 15.55% (30 nm) and 41.47% (100 nm), respectively. This result reflected the diffusion ability of passing through the membrane fenestrate from the 12-well plates to the inside of Millipore transwell. The larger liposome was weaker than the smaller one. And the two drugs addition ways of 100 nm VCR-lip showed an obvious difference. The poor diffusion ability of 100 nm VCR-lip could lead diffusion nonuniform and the high local concentration lead abundant of the cell death.

### Diffusion in the crosslinked agar

**Figure 5:**
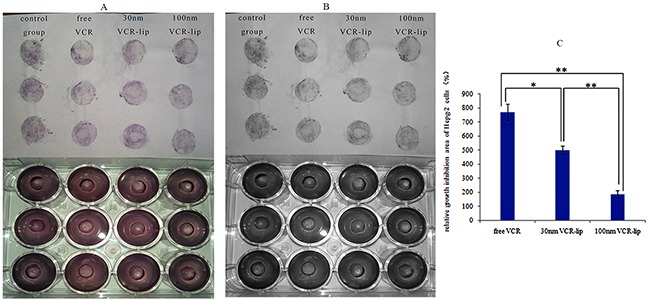
The cells growth inhibition area of different VCR-Lip and free VCR **(A)** The RGB of agar plate and transfer diagram. **(B)** The grey-scale map of agar plate and transfer diagram. **(C)** The relative growth inhibition area of HepG-2 cells.

To further proved the different diffusion ability of the free vincristine molecular and VCR-Lip with different particle size distribution in the tree-dimensional crosslinked medium [31,32], the size of HepG-2 cells inhibition zone in agar plate was compared. Figure 5A and 5B was the results of control group, free VCR, 30 nm VCR-Lip and 100 nm VCR-Lip diffusion and permeation from the oxford cup into agar to kill the HepG-2 cells. The cell inhibition area of free VCR solution, 30 nm VCR-Lip and 100 nm VCR-Lip appears a decreasing trend. The relative growth inhibition area of HepG-2 cells (Figure 5B) in turn is 769.23±59.84%, 499.41±29.77% and 185.49±27.66%. This result meant the diffusion ability was decreasing as the particle size increased. The results of diffusion in agar showed the ability of diffusion and permeation in the cross-linked agar from strong to weak was as below: free drug, 30 nm VCR-Lip and 100 nm VCR-Lip. In the other word, reduced the particle size could improve the diffusion ability in the crosslinked medium.

### Diffusion effect on apoptosis

**Figure 6:**
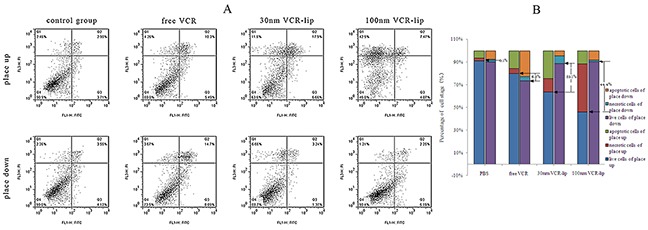
The influence of different placement methods for cells plated slide on cell apoptosis **(A)** The two-dimensional scatter diagram of flow cytometry. **(B)** The percentage of cell stage in each group.

The different placement methods of cell plated slide could affect the contact between formulations and cells. Figure 6 was the apoptosis rate of different formulations after incubation with the HepG-2 cell plated slide for different placement methods. The normal survival cell rate of the placed upwards control group, the placed down control group, the placed down 30 nm VCR-Lip group and the placed down 100 nm VCR-Lip group was 90.1%, 90.0%, 88.7% and 90.4%, respectively. These groups showed a lower value of cytotoxicity. However, for placed down free VCR group this rate was 73.5%. These values proved when place down the cell plated slide, the nanocarriers cannot diffuse to the cell side of slide and act with cells, but the free VCR could. This conclusion could be proved by the difference of cell survival rate between the place up and place down culture group (Figure 6B). This value of each group was 6.5% (free VCR), 25.1% (30 nm VCR-Lip), 44.4% (100 nm VCR-Lip). The difference value was increased along with the enlarge of nanoparticle size (diffusion ability). The left upper quadrant of 100 nm VCR-Lips group was obvious increased, for the local high concentration caused the cytoclasis. The region of 30 nm VCR-Lips group was significantly reduced (Figure 6A). This results further validated the larger particle size liposome had a weaker diffusion ability, which led to the significant difference after changing the placement methods. However, the small molecular VCR could easily diffuse to reaching uniformity in the culture solution and act with the cell on the slide. This meant that reduced the particle size of carriers could increase the diffusion ability and improve the contact opportunity of cells and carriers.

### The different diffusion ability influenced cell uptake

**Figure 7:**
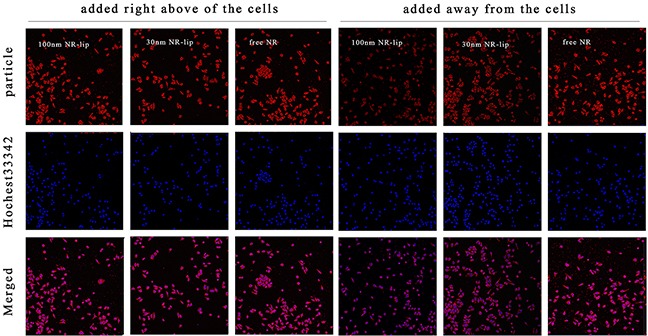
The cell uptake of different dyestuff added methods for free NR, 30 nm NR-lip, and 100 nm NR-Lip with CLSM

To investigate the diffusion behavior in the horizontal direction, we used two addition methods of fluorescein: one was adding fluorescein (or fluorescence labeling liposome) right above of the cells; the other was adding fluorescein (or fluorescence labeling liposome) at the position which was away from the cells. Figure 7 was the results of cell uptake for free NR, 30 nm NR-lip, and 100 nm NR-lip. The fluorescence intensity of each fluorescein added right above cells group was increased with the increasing of formulation particle size. But for groups with fluorescein added a distant away from the cells, the fluorescence intensity was significantly weakened with the particle size increasing. Moreover, the molecular fluorescein added way did not affect cell uptake. This result was due to the free fluorescein could diffusion uniform in a short time. While, the fluorescence labeling liposome needed long time to diffusion and acted with cells. Thus, we had a conclusion that the diffusion ability of the larger particle size liposome is weaker than the smaller one in horizontal direction and *in vitro*.

### The penetration into the tumor spheroids

**Figure 8:**
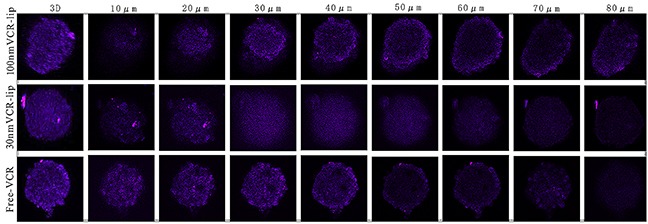
The different particle size Cy5. 5-Lip and free Cy5.5 penetrated into the tumor spheroids

Figure 8 was the CLSM results of the free Cy5.5, 30 nm Cy5.5-Lip and 100 nm Cy5.5-Lip penetrating through the tumor spheroids *in vitro*. The free Cy5.5 and 30 nm Cy5.5-Lip encapsulated fluorescein had strong penetration ability in tumor spheroids. And the penetration ability of 30 nm Cy5.5-Lip was even stronger than the free Cy5.5. We inferred this reason was that the diffusion ability of fat soluble Cy5.5 fluorescein was weak in the culture solution. The free Cy5.5 fluorescein was more easily to accumulate on the surface cells of tumor spheroids, before it penetrated into the inside of tumor spheroids. Because of the larger size Cy5.5-Lip was more difficult to penetrate into the inside of tumor spheroids than the smaller one. In this way, the larger Cy5.5-Lip only showed deeper dying on the outer layer of tumor spheroids. The tumor spheroids penetration test result shown that the small particle size Cy5.5-Lip was easier penetrating into the inside of tumor spheroids, for the higher diffusion ability of it.

### Anti-tumor effect

**Figure 9:**
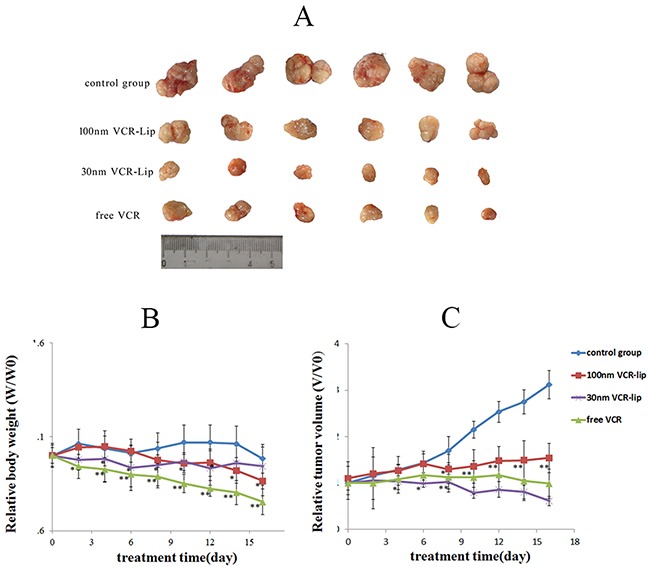
The anti-tumor effect of free VCR, 30 nm VCR-Lip and 100 nm VCR **(A)** The tumor of treatment groups and control group. **(B)** The relative body weight of each group. **(C)** The relative tumor volume of each group.

**Figure 10:**
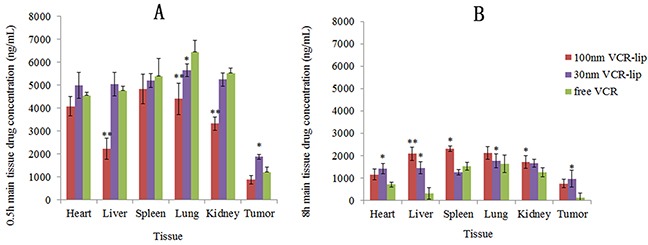
The VCR concentration in major tissues **(A)** The drug concentration of main tissues at 0.5h. **(B)** The drug concentration of main tissues at 8h.

The weight change of mice could reflect the toxicity of treatments [33] and the anti-tumor effect was reflected by the changes of tumor volume [30]. As shown in Figure 9B, the relative body weight of each treatment group decreased. The most significantly declined group was the free VCR group (0.753±0.081, m/m_0_). The relative body weight of 30 nm VCR-Lip group decreased during the early stage of treatment, but increased in the advanced stage. The body weight of 100 nm VCR-Lip group was in the opposite trend with the 30 nm VCR-Lip group. The results indicated the free VCR and 30 nm VCR-Lip was more easily to enter into the tissues. For the stronger diffusion ability from blood to tissue, they showed obvious weight loss in the early stage. However, with the tumor progression, the weigh was loosed by tumor cachexia. Because of the high diffusion ability and cell affinity, more 30 nm VCR-Lip could enter into the tumor tissue and cells to play the antitumor effect. Although the free VCR had sufficient diffusion ability, it was metabolized and excreted out of body very quickly, so the antitumor effect of free VCR was weaker than the 30 nm VCR-Lip. For the larger VCR-Lip, they were more difficulty to diffuse into major organs or tissues [31], and most of them were swallowed by the phagocytes [33] in the liver and spleen. As shown in Figure 10B, the VCR concentration of 100 nm VCR-Lip in liver and spleen was relatively higher than the free VCR and 30 nm VCR-Lip. The relative tumor volume change was shown in the Figure 9C; the trend of 30 nm VCR-Lip group was consistent with the free VCR group, and both of them were decline. However, the relative tumor volume of 100 nm VCR-Lip group and control group was increased. The final tumor inhibition rate shown in Table 2 also was given the same results. There was significant difference between the 30 nm VCR-Lip and free VCR group (P<0.05). On contrary, the free VCR group and 100 nm VCR-Lip had an extremely significant difference (P<0.01), as well as 30 nm VCR-Lip and 100 nm VCR-Lip. In summary, the anti-tumor effect showed that the smaller VCR-Lip had strong antitumor effect *in vivo* same as the free VCR and the lower toxicity, because of the stronger diffusion ability.

**Table 2:** The tumor inhibition rate of 100 nm VCR-Lip, free VCR and 30 nm VCR-Lip treatment groups

Preparation	100nm VCR-Lip	Free VCR	30nm VCR-Lip
Tumor inhibition rate (%)	51.53±10.23^**^	71.24±8.15^*^	79.31±9.56

^“*”^ mean the p value of tumor inhibition rate between the free VCR group and 30 nm VCR-Lip was less than 0.05; ^“**”^ mean the p value of tumor inhibition rate between the 100nm VCR-Lip group and the other two groups was less than 0.01, $\bar{x}\pm s$, n=6.

### Tissue distribution *in vivo*

Figure 10 was the major tissue distribution of free VCR, 30 nm VCR-Lip and 100 nm VCR-Lip group after tail vein injection for 0.5 h (Figure 10A) and 8 h (Figure 10B). The VCR concentration in various tissues of the free VCR group was higher at 0.5 h, but the VCR concentration in tumor was lower than the 30 nm VCR-Lip group and higher than 100 nm VCR-Lip. For other tissues, 100 nm VCR-Lip group was the lowest. After distribution and excretion for 8 h, the VCR concentration in various tissues for each group decreased (Figure 10B). The VCR concentration of 100 nm VCR-Lip group in various tissues was relatively higher than other groups. But in tumor, the VCR concentration of 30 nm VCR-Lip was the highest. The results of tissue distribution determination showed that with the decreasing of particle size, the quantity of drug entering into tissues increased in the short time. Because of the good diffusion ability of small molecule and smaller particle size VCR-Lip. As time went on, the drug accumulation in tissues of free drug showed obvious decline. And the 100 nm VCR-Lip obviously accumulated in the tissues which contained a large number of phagocyte cells [34]. For the 30 nm VCR-Lip group, the VCR concentration in the major tissue was lower than the 100 nm VCR-Lip group and close to the free VCR group. In tumor, the 30 nm VCR-Lip group showed stronger diffusion ability and better retention effect for the EPR effect. The tissue distribution results verified the viewpoint that decreasing particle size of nanocarriers could increase their diffusion ability which leaded to the closer distribution behavior with the free drug, and then they could diffuse into tumor tissue from the gap of angiogenesis. In addition, the smaller liposome also kept characteristics of liposome such as the strong retention effect [35], high cell affinity and modifiability.

## MATERIALS AND METHODS

### Reagents

Dipalmitoyl phosphatidylcholine (DPPC), 1,2-distearyl-sn-glycero-3-phosphoethanolamine -N- [methoxy(polyethylene glycol)-2000] (DSPE-PEG_2000_) and Monostearoyl phosphatidylcholine (MSPC) were purchased from Xi”an ruixi Biological Technology Co., Ltd, China. Vincristine sulfate (VCR, purity >99%) was bought from Min Sheng pharmaceutical group Co., Ltd, China. Dulbecco's Modified Eagle Media (DMEM) was bought from Gibco by Thermo Fisher Scientific, China. Agarose (low gelling temperature), Hochest33258, Cy5.5 and Nile red was bought from Aldrich of Sigma Co., Ltd, USA. Trypsin was purchased from solarbio. Fetal bovine serum (FBS) was bought from Zhejiang Tianhang Biotechnology Co., Ltd, China. Thiazolyl Blue Tetrazolium Bromide (MTT), Annexin V-FITC/PI Apoptosis Detection Kit was bought from GL Biochem Co., Ltd, Shanghai, China.

### Cells and animals

HepG-2 cells were kindly provided by the Drug Metabolism Department (Beijing Institute of Pharmacology and Toxicology). Nude mice (18-22g, ♀) were purchased from Vital River Laboratories (Beijing, China). All animal experiments were performed under a protocol approved by Henan laboratory animal center.

### Formulation and characterization of VCR-Liposome

#### Formulation of blank liposome

100 nm liposome was prepared [36,37] as following: DPPC: MSPC: DSPE-PEG_2000_ in a mass ratio of 85:10:5 were dissolved in chloroform, the solution was dried by solvent evaporation and formed the thin film on drying by vacuum desiccators. Then the phospholipids were hydrated to become multilamellar vesicles (MLVS) by the pH4.0 sodium tartaric buffer for 30 min. These MLVS were sonicated with a cell crusher to obtain small unilamellar vesicles. The 30 nm liposome was prepared [38] as following: The phospholipids (same mass ratio with the 100 nm liposome) were dissolved in absolute alcohol at 50°C and pH4.0 sodium tartaric buffer were added into this solution. The alcohol was removed by solvent evaporation. This lipid solution was sonicated with a cell crusher until the lipid solution become clear and transparent. The residual alcohol was removed by dialysis which molecular weight cutoff was 100, 000 Daltons. The final concentration of phospholipids (100 nm and 30 nm liposome) was 100 mg/ml.

#### VCR encapsulated liposome

The pH gradient active loading method [39] was used to contain VCR into liposome. The VCR and sucrose was dissolved in the pH4.0 sodium tartaric buffer. Then the blank liposome was added into this solution, and the sodium carbonate solution (0.3 M) was used to regulate the pH to 7.4-7.5. The theoretical drug loading was about 2 mg/ml.

### Characterization of VCR-Liposome

#### Particle distribution and morphology of liposome

The particle distribution of these two liposomes was determined by photo correlation spectroscopy (Nanophox, Sympatec GmbH, Germany) at 25°C. The VCR-Lip was diluted 20 times by water.

The morphology of two kinds of VCR-Lip was observed by a transmission electron microscope (TEM, HITACHI, H-7650, Japan). Briefly, the VCR-Lip was diluted 35 times by water and dropped it on copper grid at room temperature. The dried copper grid was stained by 2% phosphotungstic acid and then observed by TEM.

#### Determination of drug loading and encapsulation efficiency

The ultra-filtration centrifugation [40] was used to determination the drug loading and encapsulation efficiency. The un-encapsulation VCR concentration was determination as following: The VCR-Lip was diluted 10 times by water and centrifuged by ultra-filtration centrifuge tube (molecular weight cutoff was 50, 000 Daltons) at 10,000 g for 15 min. The filtrate in tube was the un-encapsulation VCR solution. The total VCR concentration sample was determined as following: The equal volume VCR-Lip and 5% twelve alkyl sodium sulfate (SDS) was mixed. The mixed solution was diluted 5 times by water and then heating it until solution clear and transparent at 50°C. The solution was used the same treatment method as the un-encapsulation VCR. The sample of un-encapsulation VCR and total VCR concentration of liposome was determination by HPLC.

The chromatographic column was ZORBAX SB-C8 (4.6×250mm, 5μm), the mobile phase was methanol and diethylamide solution (pH7.0) and the proportion was 70:30. The UV (Hitachi L-2400) detection wave length was 297 nm, the sample volume was 20μL, the column temperature was 30°C.

The entrapment efficiency (EE %) was calculated as the following equations:

$$\text{EE}\%=\frac{c_{\text{total drug}}-c_{\text{free drug}}}{c_{\text{total drug}}}\times 100\%$$ (3)

Where C_total drug_ and C_free drug_ were the total drug concentration and the un-encapsulation free drug.

### The turbiscan stability index of free VCR, 100 nm and 30 nm VCR-Lip during dilution

Three sample bottles of turbiscan [41] was respectively added 900 μL PBS. Then 100 μL of 30 nm VCR-Lip, 100 nm VCR-Lip and 2 mg/ml free VCR (dissolution by PBS) was respectively inject into the bottom of the sample bottles. Taking the other three sample bottles, each bottle was added 900 μL PBS and 100 μL VCR-Lip or free VCR, mixing the solution. Then the last bottle was added 1000 μL PBS as blank control group. All of the samples were scanned by turbiscan Lab^R^ at 37°C. The scanning time was 0 h, 0.5 h, 0.75 h, 1 h, 2 h, 4 h, 8 h, 12 h, 16 h, 20 h, 24 h, 30 h, 36 h, and 48 h.

### The effect of diffusion on cytotoxicity

**Figure 11:**
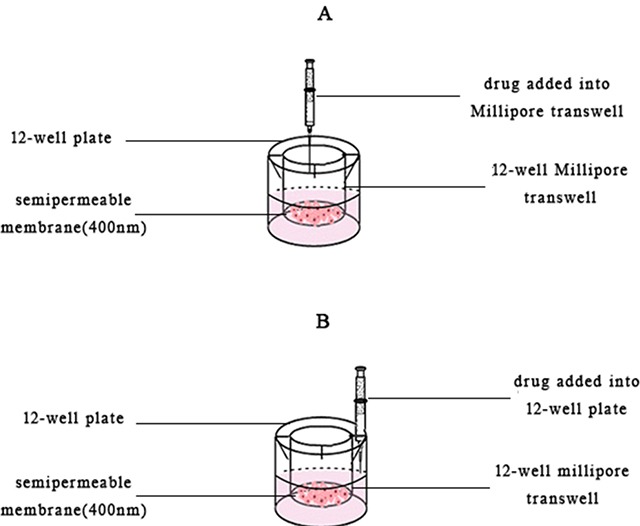
The schematic diagram of drug added methods **(A)** Added the drug into the Millipore transwells. **(B)** Added the drug into 12-well plate.

150 μL DMEM suspension contained 1.5 × 10^6^ HepG-2 cells was added in the 12-well Millipore transwell [42] and 825 ml DMEM culture medium was added in the 12 well-plate. The plate was incubated overnight (Figure 11). Then the tanswells were divided into two groups. One was added 25 μL free VCR (2mg/mL), 30 nm VCR-Lip, 100 nm VCR-Lip (n=3) was respectively added into the tanswell (Figure 11A), and the other groups was respectively added into the bottom of 12-well plate (Figure 11B). The plate was incubated for 24h, followed by add 100 μL MTT (5mg/mL) and continued to incubate for 4 h. The bottom membrane of each tanswell was taken off and dissolved by 0.4 ml DMSO. Optical absorbance was determined at 492nm with microplate reader. The cell inhibition rate was calculated as the follow equation (4).

$$\text{Cell inhibition rate}=\left(1-\frac{{c-c}_{b}}{c_{0}}\right)\times 100\%$$ (4)

Where C was the optical absorbance of drug experimental groups, C_b_ was the optical absorbance of zero adjusting, C_0_ was the optical absorbance of control group.

### Diffusion in the agar plate

**Figure 12:**
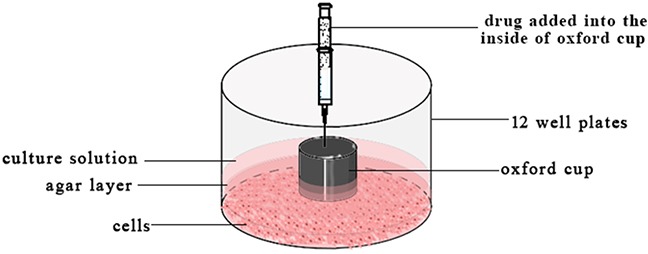
The schematic diagram of diffusion in the agar plate

2 × 10^5^/well HepG-2 cells suspension was added in the 12 well-plate and incubated overnight. The supernatant of each well was discarded and added 0.3 mL 2% (w/w) hot agar–DMEM [43] solution. When the agar was coagulation, the oxford cups (internal 5.8mm, external diameter 7.90 mm) were put on the agar plate. And 0.2 mL agar solution was added outside of the oxford cups to fix it. Then 0.3 mL DMEM culture medium was added in the outside of the oxford cups and 0.1 mL of 0.2 mg/mL free VCR, 30 nm VCR-Lip, 100 nm VCR-Lip, PBS was respectively added into the inside of oxford cups (n=3) as Figure 12. The plate was incubated for 24 h at 37°C, followed by removing the oxford cups. Discard the supernatant and washed twice by PBS. Each well was added 270 μL DMEM culture medium and 30 μL MTT and incubated for 4 h at 37°C. Then the plate was washed twice by PBS and measures the diameter of each cells inhibition zone. The agar was taken out and put on the white cardboard to transfer the print of inhibition zone. The relative growth inhibition area of HepG-2 cells (RA) as follow (5).

$$\text{RA}=\frac{S-S_{0}}{S_{0}}\times 100\%$$ (5)

Where S is the inhibition area of drug added group, S_0_ is the inner ring area of oxford cup.

### The influence of diffusion ability on cell apoptosis

**Figure 13:**
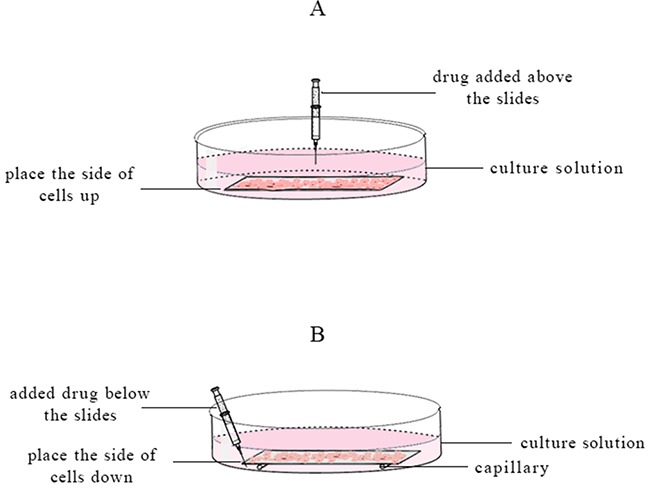
The schematic diagram of different slide placement and drug added methods **(A)** The cells side of slides was placed up and added drug on it. **(B)** The cells side of slides was placed down and added drug below it.

1 mL HepG-2 cells suspension contained 5 × 10^6^ cells was coated on the slide and incubated for 8 h. These slides were divided two groups. In one group, the cells side of slides was placed upside down and put on the capillaries (diameter 2 mm) which were adhered to the bottom of culture dish by agar. Another group, the cells side of slides was placed up and put on the bottom of dish without capillaries. Then 9.50 ml DMEM culture medium was added in the dishes. The slide in the place up group was respectively added 0.50 ml 2 mg/ml free VCR, 30 nm VCR-Lip, and 100 nm VCR-Lip above the slides (Figure 13A). The slide in place down group was added 0.50 ml 2 mg/ml free VCR, 30 nm VCR-Lip, and 100 nm VCR-Lip below slides (Figure 13B). After cultured for 24 h, the cells were stained by Annexin-V/PI and the apoptosis was determination by FAC Scan flow cytometry.

### The different diffusion ability effect on cell uptake

The fluorescent labeling Nile red lip liposome (NR-Lip) was prepared. The phospholipids proportion of NR-Lip was same with the blank 30 nm and 100 nm liposome, and then added 240 μL 0.25 mg/mL Nile red fluorescein. The following formulation procedure was same as the 30 nm and 100 nm blank liposome. The final concentration of Nile red was 10 μL/mL.

0.5 mL HepG-2 cells suspension contained 4 × 10^4^ cells was added into the laser confocal culture dish and incubated for 4 h, then added 1.5 ml DMEM culture medium. These cells were incubated overnight and discard the old culture solution. Each dish was added 1950 μL DMEM and respectively 50 μL free Nile, 30 nm NR-Lip and 100 nm NR-Lip. One group added the NR-Lip at the right above of the cells and the other group was added the NR-Lip away from the cells. The culture was incubated for 4 h and then using PBS wash the free Nile red and ploy formaldehyde fixed cell. The nucleus was stained by the Hochest33342 and the cell uptake was observed by the laser scanning confocal microscope (UltraVLEW Vox, PerkvnElmer, USA).

### Diffusion in the tumor spheroids

The prepared method of Cy5.5 fluorescent labeling liposome (Cy5.5-Lip) was same as the NR-Lip and the final concentration of Cy5.5 was 0.1 mg/mL.

1 × 10^7^ HepG-2 cells were added in the 6 well-plate and incubated the cells until they closely connected growth. The cell layer was divided into square with 1-2 mm side length by capillary. The small cells layer was fall off by digestion and transfer the cells into the 2% agar coated 96-well plate. The culture was incubated until the cells become tumor spheroids [44]. The free Cy5.5 (0.1 mg/mL), 30 nm Cy5.5-Lip (0.1 mg/mL) and 100 nm Cy5.5-Lip (0.1 mg/mL) was respectively diluted by DMEM culture medium. The dilution concentration of Cy5.5 in each group was 12.5 μL/mL. The culture medium in the 96-well plate was removed and added each diluted dyestuff 200 μL in the 96-well plate. The tumor spheroids were stained for 30 min. Then the tumor spheroids were washed by PBS and fixed by 4 %(w/v) poly formaldehyde. The tumor spheroids was scanned at different layers(10 μm/layer) by the laser scanning confocal microscope.

### *In vivo* anti-tumor efficacy

The HepG-2 tumor–bearing female nude mice (18-22 g) were used to evaluate the anti-tumor efficacy *in vivo* [45]. Each mouse was subcutaneously injected with 0.2 ml of cell suspension containing 1 × 10^7^ HepG-2 cells on the root of right arm. When the volume of tumors was about 200-300 the mice were randomly divided into 3 treatment group and 1 control group (n=6). Each mouse was treated every 72 h by tail vein injection (1.25 mg/kg dose). The control group mice were given 0.625 mL/kg normal saline. The tumor volume was measured every two-day and calculated the volume based on the equation (6). The mice were weighed every day during the period of treatment. After 16 days, the mice were sacrificed and weighed the tumor of each mice. The relative body weight and tumor volume was calculated as the following equations (7, 8).

$$V=\frac{\left(a\times b^{2}\right)}{2}$$ (6)

Where a was indicated the length and b was indicated the width of the tumor.

$$\text{Relative body weight}=\frac{m_{t}}{m_{0}}$$ (7)

Where m_t_ was the t day's body weight and m_0_ was the initial body weight of mice.

$$\text{Relative tumor volume}=\frac{V_{t}}{V_{0}}$$ (8)

Where V_t_ was the t day's tumor volume and V_0_ was the initial tumor volume of mice.

### Tissue distribution studies

Firstly, we randomly divided 24 tumor bearing female nude mice (HepG-2) into three groups as the drug experimental groups (n=6, free VCR group, 30 nm VCR-Lip group, and 100 nm VCR-Lip group) and a blank control group. After given the different formulations 0.5 h and 8 h, three mice of each group were respectively sacrificed, and then the heart, liver, spleen, lung, kidney and tumor were removed to determination the VCR concentration in each tissue.

### Determination of the drug concentration of tissues

Each tissue and triple normal saline was homogenized together. 10 μL vinblastine sulfate (200 ng/mL) was added in 100 μL tissue homogenate and 100 μL tert-butylmethyl as internal standard solution, then the mixture was full mixed by vortex for 30 s and taken the centrifugal supernatant at 14,000 r/min for 5 min. The supernatant was concentrated and the residue was dissolved in 100 μL mobile phase.

The Agilent 1200-6410 Triple Quad LC/MS system was used to analyzed the sample of each tissue. Agela Venusil XBP C18 (2.1mm×50mm, 3μm) was used to separation the sample. The column temperature was held at 30 XBPAgimobile phase consisted was methanol-5mM ammonium acetate solution (solution A, 30:70, pH9.8, V/V) and methanol-5mM ammonium acetate solution (solution B, 90:10, pH9.8, V/V). The gradient elution program was showed in Table 3 and the sample volume was 10 mL. The total analysis time was 5 min.

**Table 3:** Program of gradient elution

Time(min)	Mobile phase A:B	Flow rate (ml/min)
0.0	40:60	0.4
0.2	0:100	0.4
1.48	0:100	0.4
1.5	40:60	0.4
5.0	40:60	0.4

The condition of mass spectrometry was as following: The detection of ions was used the positive ionization mode and the ion detection method were used multiple reaction-monitoring mode. The detection object was VCR (m/z, 825.4→807.2) and internal standard (m/z, 811.3→223.9). The Capillary voltage was 4000 V and the fragmentation voltage was 180 V. The collision energy was 40 eV for VCR and 45 eV for internal standard. The nitrogen was used as drying gas and nebulizing gas. The gas flow was 9 L/min [46] and the source temperature was 350°C.

### Statistical methods

Descriptive statistics included the mean and standard error except particularly outlined. The statistical significance of the difference was tested using Student's t-test. ^“*”^ mean the *P*-value less than 0.05 which was considered to be significant and ^“**”^ mean the *P*-value less than 0.01 which was considered to be highly significant.

## CONCLUSION

This study used the larger and smaller particle size liposome and the free drug or fluorescein to demonstrate the different diffusion ability *in vitro* and *in vivo*. All the results showed that the free drug have the strongest diffusion ability which could quickly diffused to uniformity in solution after dilution. For the drug loaded liposome, the diffusion ability of larger size liposome was weaker than the small one. To a certain extent, the particle size influenced the diffusion to the surface of the cells *in vivo* and *in vitro*. Thus influenced further inhibition of the tumor cells. The water soluble drugs had better diffusion ability in solution. However, the lower membrane permeability of them leaded to the poorer cytotoxicity when acting on tumor cells. And the lipid soluble drugs have the lower solubility in aqueous solution which leads to the lower concentration. Then the free drugs need larger dose to inhibit tumor cells comparing with the liposome encapsulated drugs with high drug loading ability, good water solubility and cell membrane penetration. On one hand the smaller liposome has better diffusion ability and higher particle concentration, which leads them easily reach tumor cells. On the other hand, they retain the characteristics of water solubility and cell penetration of liposome. In conclusion, reducing the particle size of liposome preserves the characteristics of liposome and strong diffusion ability, which results in the smaller liposome having sufficient quantity and ability to diffuse into tumor tissue from the tumor angiogenesis. These nanocarriers entering tumor tissue could also further play the active targeting effect if they are modified. Therefore, increasing the diffusion ability is the premise to enhance the active target and passive target effect of nano drug delivery system.

## Abbreviations

VCR: vincristine
VCR-Lip: vincristine-liposome
Cy5.5-Lip: cyanine5.5 NHS ester labeled liposome
TEM: transmission electron microscope
MTT: methyl thiazolyl tetrazolium
RGB: red-green-blue
NR: Nile red
